# Supplementary material for: In Situ Regeneration of Copper-Coated Gas Diffusion Electrodes for Electroreduction of CO2 to Ethylene
Source: Materials (Basel). 2021 Jun 9;14(12):3171. doi: 10.3390/ma14123171 (PMC8228262; doi:10.3390/ma14123171)
Supplement: Supplementary file 1 [file materials-14-03171-s001.zip › materials-1223820-SI.pdf]

Electronic supplementary information

In Situ Regeneration of Copper-Coated Gas Diffusion Electrodes for Electroreduction of CO_2 to Ethylene

Magdalena Bisztyga-Szklarz [1], Krzysztof Mech [1*], Mateusz Marzec [1], Roberts Kalendarev [2], and Konrad Szaciłowski [1*]

[1] AGH University of Science and Technology, Academic Centre for Materials and Nanotechnology, al. A. Mickiewicza 30, 30-059 Krakow, Poland; mbs@agh.edu.pl (M.B.S.) kmech@agh.edu.pl (K.M.); mmarzec@agh.edu.pl (M.M.); szacilow@agh.edu.pl (K.S.)

[2] Institute of Solid State Physics, University of Latvia, Kengaraga Street 8, LV-1063 Riga, Latvia; robert.kalendarev@cfi.lu.lv

* Correspondence: kmech@agh.edu.pl (K.M), szacilow@agh.edu.pl (K.S.)

Table 1. Results of EDS analysis.

Site	Element	Wt %	At %	Error %
1	C	52.5	70.1	7.6
	O	14.1	14.1	10.4
	F	11.4	9.6	9.8
	K	4.0	1.7	2.6
	Cu	18.0	4.5	3.1
2	C	50.7	11.0	10.8
	O	10.1	5.9	10.1
	F	6.4	1.7	3.4
	K	3.7	8.0	3.0
	Cu	29.1	11.0	10.8
3	C	53.3	69.9	7.4
	O	15.2	15.0	10.2
	F	11.8	9.8	9.7
	K	3.2	1.3	2.6
	Cu	16.5	4.1	3.1
4	O	13.2	31.4	9.2
	F	8.4	16.9	9.1
	K	12.4	12.1	3.3
	Cu	66.0	39.6	2.4
5	O	33.2	61.2	9.4
	K	26.8	20.2	2.2
	Cu	40.0	18.6	2.4
6	O	36.1	64.8	9.1
	K	22.2	16.3	2.4
	Cu	41.7	18.8	2.4
7	O	33.2	61.2	9.4
	K	26.8	20.2	2.2
	Cu	40.0	18.6	2.4
8	O	36.1	64.8	9.1
	K	22.2	16.3	2.4
	Cu	41.7	18.8	2.4
